# Androgen receptor as potential therapeutic target in metastatic endometrial cancer

**DOI:** 10.18632/oncotarget.10334

**Published:** 2016-06-30

**Authors:** Ingvild Løberg Tangen, Therese Bredholt Onyango, Reidun Kopperud, Anna Berg, Mari K. Halle, Anne M Øyan, Henrica M.J Werner, Jone Trovik, Karl Henning Kalland, Helga B. Salvesen, Camilla Krakstad

**Affiliations:** ^1^ Centre for Cancer Biomarkers, Department of Clinical Science, University of Bergen, Norway; ^2^ Department of Gynecology and Obstetrics, Haukeland University Hospital, Norway; ^3^ Centre for Cancer Biomarkers, Department of Clinical Medicine, University of Bergen, Norway; ^4^ Department of Microbiology, Haukeland University Hospital, Norway; ^5^ Centre for Cancer Biomarkers, Department of Biomedicine, University of Bergen, Norway

**Keywords:** androgen receptor, endometrial cancer, biomarker, survival

## Abstract

**Purpose:**

The expression and involvement of estrogen (ER) and progesterone receptor (PR) is extensively studied in endometrial cancer. Androgen receptor (AR) is a hormone receptor less studied in female cancers, and we here aim to investigate the expression level of AR in endometrial cancer precursor lesions, primary tumors and metastases, and its potential as therapeutic target.

**Results:**

Expression of AR was observed in 93% of hyperplasias, but only in 41% of non-endometrioid tumors. Compared to estrogen and progesterone receptor AR is more commonly expressed in metastatic lesions, and AR status is discordant in primary and metastatic lesions in a large proportion of cases. AR protein level was significantly associated with survival (*P* < 0.001), and a calculated AR to ERα ratio identified a subgroup of patients with particular poor outcome. The anti-androgen enzalutamide may have a growth inhibitory effect in endometrial cancer cells based on experiments with primary endometrial tumor cells.

**MATERIALS AND METHODS:**

718 primary endometrial cancers and 298 metastatic lesions (from 142 patients) were investigated for expression of AR in relation to survival, clinical and histopathological data. Protein levels were investigated by immunohistochemistry and reverse phase protein array; mRNA levels by DNA oligonucleotide microarray. The effect of androgen stimulation and inhibition was tested on primary endometrial tumor cells.

**Conclusions:**

A large proportion of metastatic endometrial cancer lesions express AR, which may be a potential target in these patients. Treatment targeting AR may be of particular benefit in patients with high AR levels compared to ERα levels.

## INTRODUCTION

Endometrial cancer is the most common gynecological malignancy in the Western countries [1]. Little progress has been made in development of treatment options over the past decades. This, combined with a rising incidence, has resulted in an increased number of deaths caused by the disease [2]. Although patients with localized disease have a good prognosis, prognosis is poor for patients with recurrence or metastatic disease at diagnosis and treatment options are few [3]. To improve treatment of this patient group, both the identification of new treatment targets, and identification of potent biomarkers to aid patient stratification are vital.

Traditionally, endometrial cancers have been divided into two groups. Type I, accounting for approximately 80% of cases, is associated with endometrioid histology, low stage and grade, good prognosis and estrogen dependency [3, 4]. Type II tumors are associated with non-endometrioid histology, high stage and grade and poor prognosis [3]. The majority of endometrial cancers are hormone dependent, and hormonal dysregulation is linked to disease development and progression [5]. The role and expression of estrogen and progesterone receptors (ERα and PR) in endometrial cancer have been extensively studied [5]. ERα and/or PR positivity in primary tumors is associated with well-differentiated lesions and more favorable prognosis [6, 7]. Hormonal therapy targeting both PR and ERα is used in treatment of endometrial cancer. The response rate to such treatments is usually low [8], however, patients expressing hormone receptors have been shown to be more sensitive to hormonal therapy [2].

Androgen receptor (AR) is a hormone receptor less studied in endometrial cancer, although a target for treatment in other cancers [9, 10]. It is expressed in several tissues, including the uterus, where its role is largely unknown [10, 11]. In males, aberrant androgen signaling is central to initiation and progression of prostate cancer [12, 13]. Treatment targeting AR or androgen synthesis is therefore one of the main therapeutic elements in patients with hormone dependent prostate cancer [9]. Recently, also in breast cancer treatment, targeting AR has been suggested beneficiary for specific subgroups of patients [10].

Given the similarities of breast and endometrial cancers, exploring AR expression in endometrial cancer might reveal new therapeutic strategies. Additionally, AR status in malignant endometrial lesions may be related to clinical phenotype and could represent a novel biomarker for prognosis.

## RESULTS

### AR is expressed in the majority of hyperplasias and primary endometrial cancer lesions

**Figure 1:**
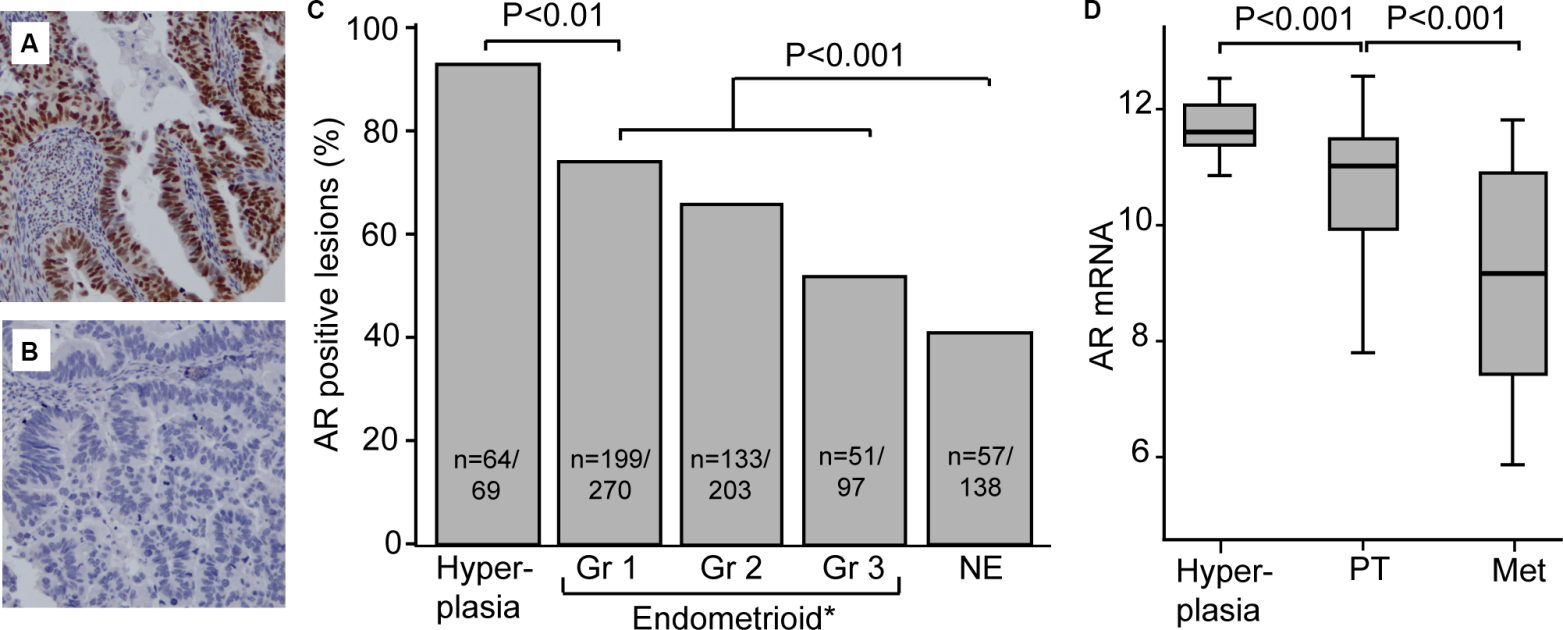
AR expression decreases with dedifferentiation AR expression in glandular cells was scored, with strong AR immunohistochemical staining shown in (**A**) and AR loss in (**B**). Both AR protein level (**C**) and mRNA (**D**) level decreased with dedifferentiation. Abbreviations: AR: androgen receptor, Gr: grade, NE: non-endometrioid, PT: primary tumor, Met: metastases, *Information on grade missing for 10 patients included in the study (7 AR positive and 3 AR negative)

The expression pattern of AR was investigated in relation to aggressiveness of endometrial cancer. AR staining was predominantly nuclear, and only staining in glandular tissue was scored (Figure 1A and 1B). Precursor lesions were included to investigate if there was a change in expression from the pre-malignant to the malignant stage. Hyperplasias had the highest level of AR expression. Of the 69 hyperplasias evaluated, 93% (64 patients) expressed AR. In primary tumors the percentage of lesions with AR expression decreased significantly from 74% to 53% from endometrioid grade 1 to grade 3 tumors respectively and further to 41% in non-endometrioid tumors (Figure 1C). This pattern was reflected when assessing mRNA levels (Figure 1D), and there was significant overlap between AR expression evaluated by IHC and AR mRNA expression (Supplementary Figure 1).

### AR is frequently expressed in metastatic lesions of endometrial cancer

**Figure 2:**
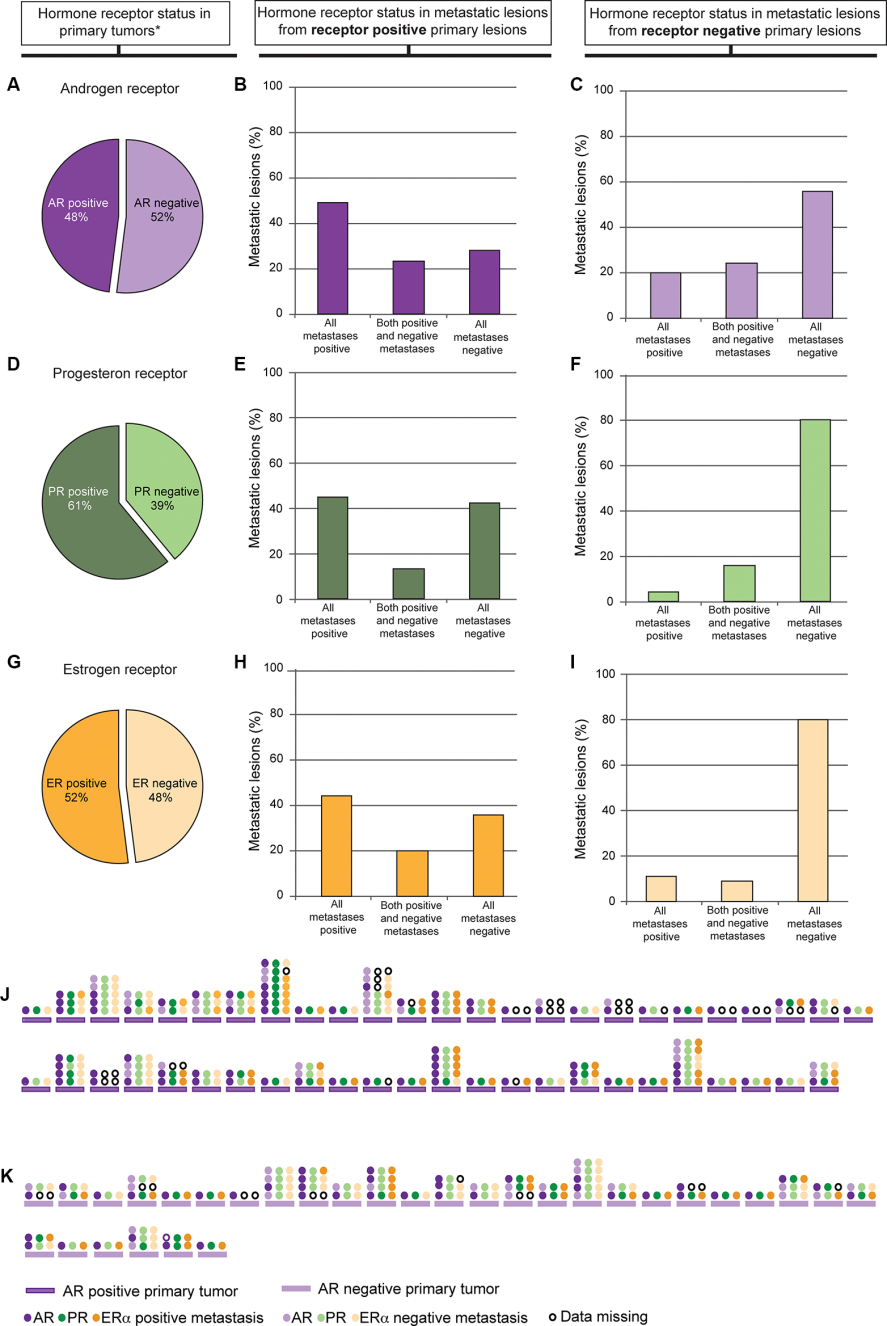
Expression of the individual hormone receptors AR, PR and ERα in metastatic lesions based on its expression in primary tumor. 48% of the primary tumors with corresponding metastatic lesions were AR positive (IHC score 1–9) (**A**). Graphs show distribution of AR expression in the metastatic lesions from AR positive primary tumors (**B**) and AR negative primary tumors (**C**). 61% of the primary tumors with corresponding metastatic lesions were PR positive (IHC score 1–9) (**D**). Graphs show distribution of PR expression in the metastatic lesions from the PR positive primary tumors (**E**) and PR negative primary tumors (**F**). 52% of the primary tumors with corresponding metastatic lesions were ER positive (IHC score 4–9) (**G**). Graphs show distribution of ER expression in the ER positive primary tumors (**H**) and ER negative primary tumors (**I**). Expression patterns in individual metastases from AR positive patients (**J**) and AR negative patients (**K**) are shown as AR (purple), PR (green) and ERα (orange) in J and K. One line of circles represents one metastasis, and shows the difference in hormone receptor expression in that specific metastasis. Only patients with one or more AR positive metastases are shown. *Only primary tumors with corresponding metastases included.

As for most cancers, metastatic endometrial cancers have high mortality and few treatment options. Characterization of metastatic lesions is therefore highly important, especially considering molecular targets with existing therapeutics. Expression was investigated in 298 metastatic lesions from 142 patients (Supplementary Table 1). We observed that a high number of the metastatic lesions express AR. For patients with corresponding metastatic lesions AR, PR and ERα was expressed in 48%, 61% and 52 % of the primary tumors respectively (Figure 2A, 2D and 2G). In metastases from hormone receptor (AR, PR or ERα) positive primary tumors, expression was retained in at least one metastatic lesion in 71% of cases for AR, 58% for PR and 64% for ERα (Figure 2B, 2E and 2H). Also 44% of patients defined to have lost expression of AR in the primary tumor expressed AR in at least one metastatic lesion (Figure 2C). For ERα and PR this was only 20% (Figure 2F and 2I). AR, PR and ERα were investigated in individual metastases to compare expression patterns. A high degree of heterogeneity in hormone receptor expression was observed both in metastases from AR positive primary tumors (Figure 2J) and AR negative primary tumors (Figure 2K). 39% of the metastatic lesions expressing AR had low expression of ERα.

### Expression of AR is associated with good survival

**Figure 3:**
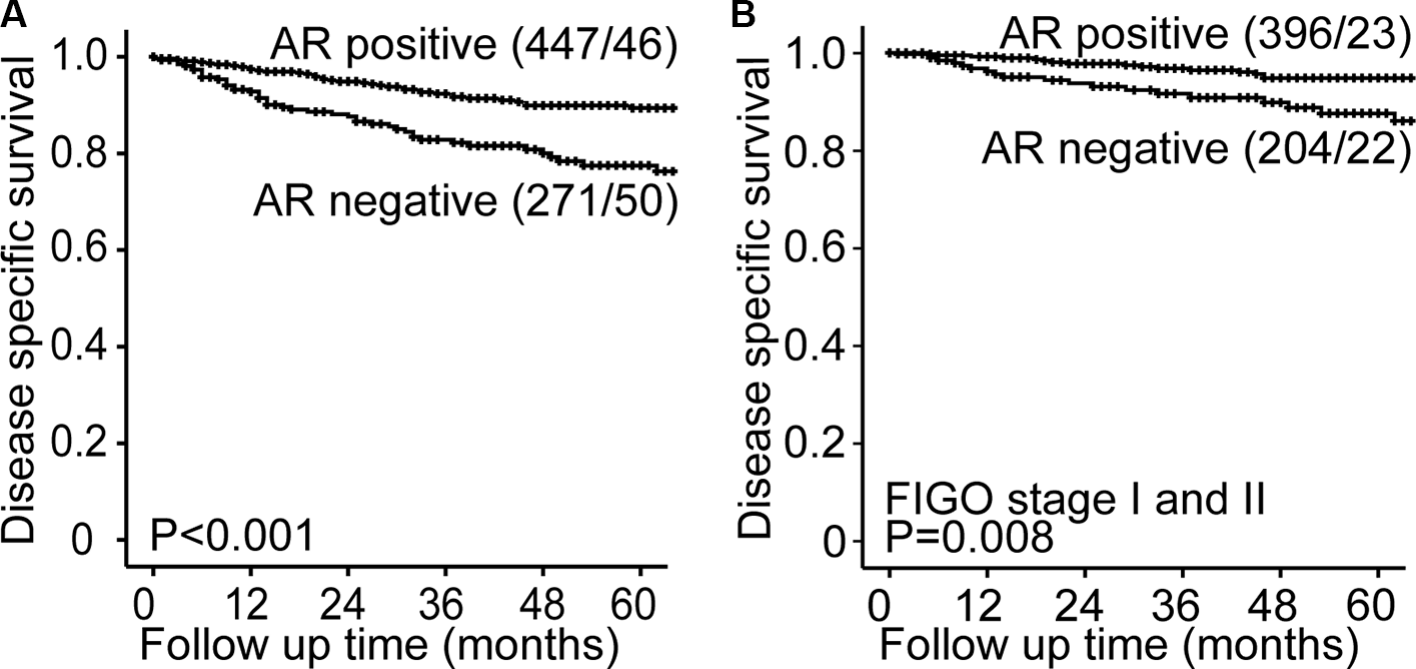
AR status predicts prognosis in endometrial cancer Endometrial cancer patients with AR expression (scoring index 1–9) have a significantly better survival than patient with AR loss (scoring index 0) both in the whole population (**A**) and in the subgroup of patients with FIGO stage I and II (**B**).

Loss of AR was significantly associated with established features of aggressive tumors, such as high FIGO stage (*P* < 0.001), non-endometrioid histology (*P* < 0.001) and high grade within the endometrioid subgroup (*P* = 0.001) (Table 1). The relation between disease specific survival and AR expression was investigated using groups with high and low expression of AR as defined in the method section. AR loss associated with shorter disease specific survival both in the whole population (Figure 3A) and within the subgroup of patients with disease confined to the uterus, FIGO stages I/II (Figure 3B). In multivariate survival analyses AR did not demonstrate independent prognostic impact when adjusting for factors with known prognostic value (age, histologic type and grade) (*p* = 0.12, data not shown), indicating that loss of AR may not add additional information regarding survival when used in a clinical setting. Still, the high number of primary tumors and metastatic lesions with intact expression of AR could point to an unexploited potential for treatment targeting AR in endometrial cancer, and it might be of particular interest in specific subgroups as observed for other cancer types.

**Table 1:** Clinico-pathological variables related to androgen receptor (AR) status in endometrial cancer patients

	AR		
Variable	Positive *n* (%)	Loss *n*(%)	*P*-value
Age			0.7
< 66	229 (63)	135 (37)	
≥ 66	218 (62)	136 (38)	
FIGO-09 stage			< 0.001
I–II	396 (66)	204 (34)	
III–IV	51 (43)	67 (57)	
Histologic type			< 0.001
Endometrioid	386 (67)	188 (33)	
Adenosquamous	4 (67)	2 (33)	
Clear cell	7 (25)	21 (75)	
Serous papillary	36 (55)	29 (45)	
Carcinosarcoma	13 (41)	19 (59)	
Undifferentiated/other	1 (7)	12 (92)	
Histologic grade*			0.001
Grade 1/2	329 (70)	140 (30)	
Grade 3	50 (53)	45 (47)	
Metastatic nodes			< 0.001
Negative	333 (65)	176 (35)	
Positive	28 (42)	39 (58)	
Ploidy			0.3
Diploid	223 (64)	128 (36)	
Aneuploid	59 (58)	43 (42)	
PR			< 0.001
Positive	391 (74)	137 (26)	
Negative	40 (26)	115 (74)	
ERa			< 0.001
Positive	390 (78)	113 (22)	
Negative	42 (23)	140 (77)	

* only endometrioid, missing information on grade for ten cases.

### High AR to ERα ratio identifies patients with particularly poor survival

**Figure 4:**
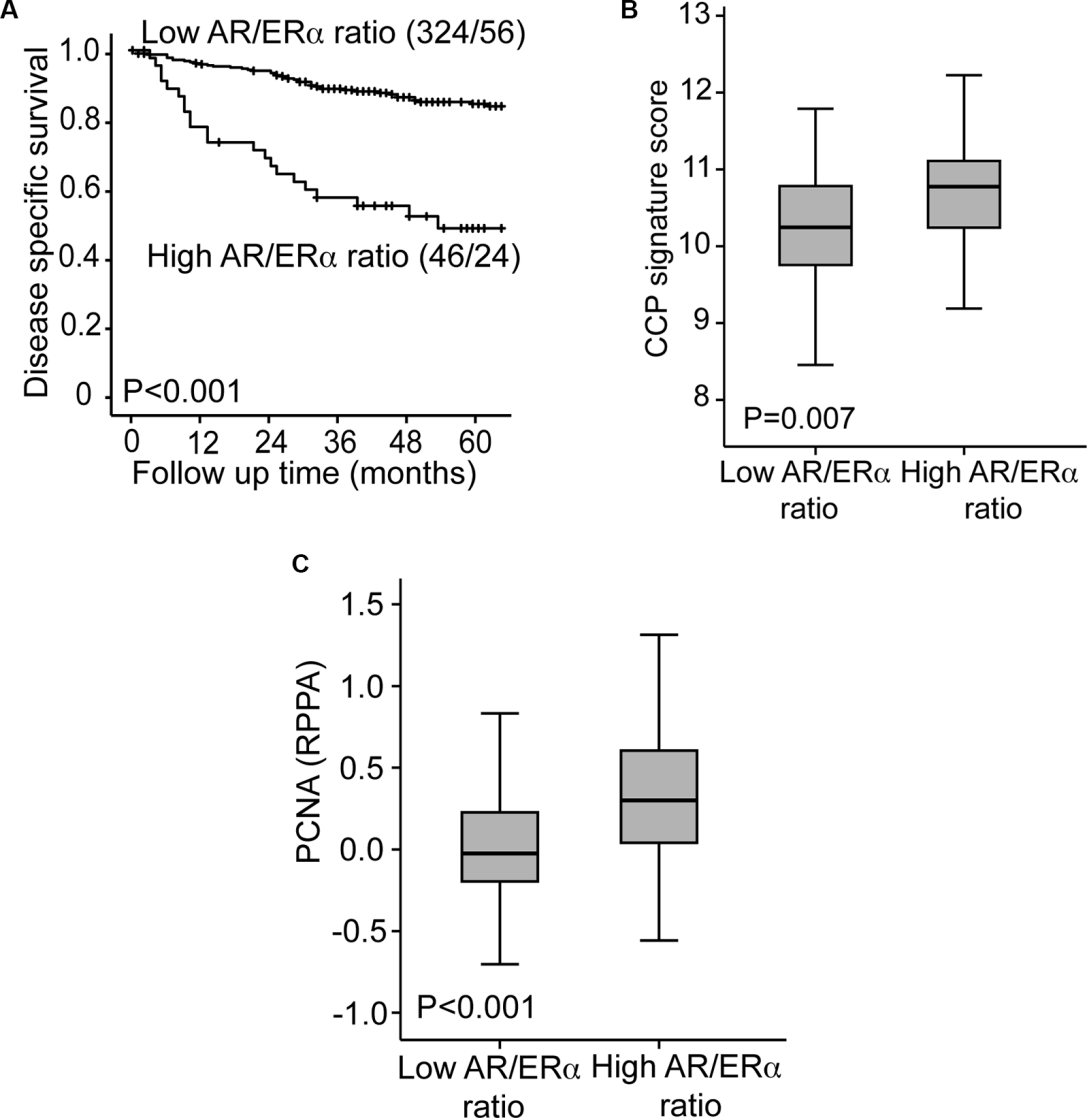
High AR to ER ratio identifies a subgroup with particularly poor survival Patients with a high AR to ERα ratio assessed by Reverse Phase Protein Array demonstrate a particularly poor disease specific survival compared to patients with a low AR to ERα ratio (**A**). The group with high AR to ERα ratio had significantly higher CCP score (**B**) and PCNA expression (**C**) compared to the group with low AR to ERα ratio.

Based on previous findings in breast cancer, we hypothesized that also for endometrial cancer, the effect of AR signaling may be influenced by the presence of ERα. Interestingly, the patients with the highest calculated AR to ERα ratio (based on RPPA data) had significantly worse survival, both in the whole population (Figure 4A) and in FIGO stages I/II (Supplementary Figure 2A). A high ratio was also significantly associated with established features of aggressive tumors (Supplementary Table 2). In a subpopulation with especially long follow up, a high AR to ERα ratio, calculated based on mRNA levels, also identified a patient group with significantly worse survival compared with patients with a low AR to ERα ratio, both in the whole population (Supplementary Figure 2B) and in FIGO stages I/II (Supplementary Figure 2C).

The underlying mechanisms involved were explored by investigating transcriptional alterations related to the high AR to ERα ratio group. In GSEA analysis several of the top ranked GO gene sets enriched in the high AR to ERα ratio group were associated with cell cycle regulation (Supplementary Table 3). This finding was supported by the significantly higher proliferation identified in patients with high AR to ERα ratio, assessed both by high cell cycle progression (CCP) score and high proliferation cell nuclear antigen (PCNA) levels (measured by Reverse Phase Protein Array) (Figure 4B and 4C).

To explore if transcriptional alterations related to the high AR to ERα ratio could suggest new targets for treatment, Connectivity map was queried for drug signatures negatively correlated with the gene expression profile of tumors with a high AR to ERα ratio. Compounds targeting phosphoinositide 3-kinase (PI3K)/mammalian target of rapamycin (mTOR) pathway were among the top ranked, along with HSP90 inhibitors, known to disrupt hormone binding and hormone receptor stability [14], the AR inhibitor Resveratol [15, 16] and a CDK inhibitor [17] (Supplementary Table 4). These findings suggest that drugs targeting proliferation and anti-androgen treatment could be potential treatment options for the high AR to ERα ratio patient subgroup in particular. Supporting this, preliminary results in short-term cultures of AR positive (defined by IHC staining of patient biopsy) primary endometrial tumor cells, proliferation was significantly inhibited by the anti-androgen enzalutamide, and there was a tendency to increased proliferation after stimulation with the synthetic androgen R1881, although not statistically significant. No effect was seen in AR negative or focal positive cells (data not shown).

## DISCUSSION

Several studies have demonstrated that ERα and PR status are important prognostic biomarkers, also predicting response to anti-hormonal therapy in endometrial cancer [6, 7]. For AR available data is more limited. It is still unsettled if AR could predict outcome and also, whether there is a role for androgen targeting drugs in endometrial cancer treatment. The published studies are few with low number of included patients, leaving inconclusive results [18–24]. In the present study, to date the largest and most comprehensive study with extensive clinical annotation and samples ranging from precursors through primary to metastatic endometrial carcinoma lesions, we find that precursor lesions and well-differentiated primary tumors have the highest level of AR, with a gradual decrease in AR level with dedifferentiation. This pattern is consistently found using different methods to assess AR levels. Our findings thus appear to be in line with previous smaller studies, reporting AR loss ranging from 11.4% in endometrioid primary tumors [25] to 79% in a study including primary tumors of different histological subtypes [26]. A significant association between dedifferentiation and AR loss has also been reported in a small study including 35 patients [27]. In this study we show that also AR is a prognostic marker in endometrial cancer as earlier well documented for ERα and PR [6, 28–30].

The extensive experience with anti-androgen treatment in prostate cancer makes it attractive for investigation also in other hormone dependent cancers. There are several available anti-androgen treatments with different mechanism of action. The anti-androgen bicalutamide inhibits binding of ligand to the androgen receptor, while enzalutamide, a second generation anti-androgen, inhibits several steps of AR activation. Inhibition of AR signaling can also be achieved through inhibition of ligand synthesis. Abiraterone inhibits the enzyme CYP17 which is necessary for androgen biosynthesis [31]. Although our findings show that AR expression is associated with low grade tumors, it may, analogous to ERα, still be a driver for tumor growth and therefore a potential therapeutic target. Our preliminary results indicate that enzalutamide may inhibit proliferation in AR positive primary endometrial cancer cells. This is an interesting observation that calls for follow-up functional studies. There are few studies investigating the effect of AR signaling on endometrial cells, and the results from these studies are contradictory. Different mechanisms have been suggested by which androgens may exert their effect on the endometrium. The proliferative effects of androgen have been linked to enhancement of EGF action [18], increased expression of genes involved in IGF-1 and Wnt signaling [19] or to endometrial cancer cell proliferation through activation of the oncogene c-myc [20]. These studies are however contradicted by the finding that androgens inhibit proliferation of endometrial cells *in vitro* [21, 22], and several studies on the weakly AR positive endometrial cancer cell line MFE-296 demonstrating growth inhibition by androgen stimulation [23, 24]. These conflicting results could imply different roles for AR at different stages of endometrial cancer and indicate that AR signaling is context-dependent. This is in line with our finding that a subgroup of patients with high AR to ERα ratio has particularly poor survival, indicating that ERα status may influence the effect of AR. Our findings are also in line with previous observations from breast cancer, where the prognosticvalue of AR is improved by combining AR and ERα status [32, 33]. Cochrane *et. al.* found that a high AR to ERα ratio was an independent predictor of disease-free and disease specific survival in breast cancer [34]. Studies are ongoing in breast cancer to investigate the effect of anti-androgen treatment in patients with AR positive tumors with loss of ERα and PR (ClinicalTrials.gov Identifier NCT00468715, NCT01889238). Similar studies in endometrial cancer patients could reveal whether AR could be a potential target also in endometrial cancer patients with high AR to ERα ratio.

Hormonal treatment targeting ERα and PR is found to be most effective if the hormone receptor is expressed [2, 35]. Since the expression of these hormone receptors is discordant between primary and metastatic lesions [6, 36], investigating the expression status also in the metastatic setting is important. We find that AR is more often expressed in metastatic lesions compared to ER and PR, but also AR expression is discordant in primary and metastatic lesions in a large proportion of cases. Since a large proportion of metastatic endometrial cancers express AR, this may serve as a potential therapeutic target for this patient group, and particularly in the group with a high AR to ERα ratio. As there are several available treatments targeting AR, further studies should be conducted to investigate their potential in treatment of endometrial cancer. This study also underlines the need to examine metastatic expression of potential therapeutic targets, as multiple studies have also done concerning other biomarkers and targets [6, 36–38], and clearly demonstrates that the primary tumor is not representative for its metastatic lesions.

## MATERIALS AND METHODS

### Patient series

A population-based patient series was prospectively collected from 2001 to 2015 and included 718 primary tumors from patients diagnosed with endometrial cancer in Hordaland County (Norway). Clinical data were collected as previously described [39] and patients were surgically staged according to the International Federation of Gynecology and Obstetrics (FIGO) 2009 criteria. Samples from precursor lesions (complex atypical hyperplasia) were available from 69 patients and metastasis from 142 patients (in total 298 metastatic lesions). Fresh frozen tissue were investigated for gene expression for 223 patients of which 163 overlapped with formalin fixed paraffin embedded tissue (FFPE). In addition protein expression was investigated in 370 patients by reverse phase protein array (RPPA) where 306 overlapped with FFPE (Supplementary Table 5).

The study has been approved by Norwegian legislation, including the Norwegian Data Inspectorate, Norwegian Social Sciences Data Services and the Western Regional Committee for Medical and Health Research Ethics (REK 2009/2315). Patients gave written informed consent.

### Immunohistochemical staining

Tissue microarrays (TMA) were dewaxed in xylene and rehydrated in graded ethanol series before microwave boiling in target retrieval buffer (pH9) for 15 minutes. Staining for AR, ERα and PR expression was performed as previously described [6, 7, 40]. The staining was evaluated using a semi-quantitative system where both intensity and area of positive tumor cells is considered. Staining intensity was graded from 0 (no staining) to 3 (strong staining), and area from 0, 1 (< 10%), 2 (10–50%) and 3 (> 50%). A staining index was calculated as the product of staining intensity and area. For AR and PR, staining index 0 was considered negative, and index 1–9 positive. For ER index 0–3 was defined as low, and 4–9 as high. The method for producing tissue microarrays (TMA) has previously been described and validated [41, 42].

### Gene expression analysis

RNA was extracted from fresh frozen tissue from 18 hyperplasias, 174 primary tumors and 42 metastases, and hybridized to Agilent Whole Human Genome Microarrays 44k (Cat. No. G4112F), according to the manufacturer's instruction. The arrays were scanned and normalized as previously described [36]. Gene Ontology (GO) gene sets enriched in different groups were investigated with Gene Set Enrichment Analysis (GSEA) using gene permutations and Significance Analysis of Microarray (SAM) as scoring method. The signature score for the cell cycle progression (CCP) signature published by Cuzik et al [43] was calculated as previously described [6]. Connectivity Map [44] was used to search for potential drugs based on differences in gene expression between the high AR to ERα ratio and the AR to ERα ratio group. 23 cases with high ratio (≥ 1.25), and the 41 cases with lowest ratio (≤ 0.95) were included in the analysis. The AR to ER ratio based on mRNA expression was calculated by dividing the AR value with ER value.

### Reverse phase protein array

Reverse Phase Protein Array (RPPA) was performed for 370 of the primary tumors as previously described [45, 46]. Slides were stained using antibodies as listed (https://www.mdanderson.org/education-and-research/resources-for-professionals/scientific-resources/core-facilities-and-services/functional-proteomics-rppa-core/index.html). Relative protein levels were determined by fitting each dilution curve with a logistic model (‘Supercurve Fitting’ http://bioinformatics.mdanderson.org/OOMPA) [47]. Expression levels of AR, ERα and proliferation cell nuclear antigen (PCNA) were extracted from the whole RPPA dataset. The AR to ERα ratio was calculated by dividing the AR value with ERα value.

### Statistical analysis

Data were analyzed using SPSS version 22 (SPSS Inc, Chicago, IL). Probability < 0.05 was considered statistical significant. Associations between groups were evaluated using the chi-square test for categorical variables and the Mann-Whitney U test for continuous variables. Univariate survival analysis was performed using the Kaplan Meier (product-limit) method. Date of primary surgery was entry date, and date of death due to endometrial carcinoma was defined as event for estimation of disease specific survival. Survival between groups was compared using the log-rank (Mantel-Cox) test. The Cox proportional hazard regression model was used to evaluate the prognostic impact of AR adjusted for other prognostic parameters. Included in the model was in addition to AR; age, histologic type and grade, all factors with known prognostic impact in endometrial cancer.

## SUPPLEMENTARY MATERIALS FIGURES AND TABLES


