# Supplementary material for: Androgen receptor as potential therapeutic target in metastatic endometrial cancer
Source: Oncotarget. 2016 Jun 30;7(31):49289–98. doi: 10.18632/oncotarget.10334 (PMC5226508; doi:10.18632/oncotarget.10334)
Supplement: Supplementary file 1 [file oncotarget-07-49289-s001.pdf]

Androgen receptor as potential therapeutic target in metastatic endometrial cancer

Supplementary Materials

MATERIALS AND METHODS

Primary endometrial cancer cell experiments

The tumor specimens were, immediately after receiving them from the operation room, minced into single cell suspension filtrated and washed with PBS. Cells were seeded in 96-well plates in phenol red free media (MEM) with charcoal stripped FBS containing 10 µM enzalutamide or 10 nM R1881 or media only as control. Experiments were performed in triplicates. These short term cultures were kept for 15 days. Effect on proliferation was determined by MTS assay (CellTiter 96 Aqueous One Solution Cell Proliferation Assay, Promega), according to the manufacturer's instruction. Absorbance was recorded at 490 nm using an ELISA plate reader (TECAN Magellan Sunrise). Hormone receptor status of tumors used for primary endometrial cancer cell experiments were determined by IHC staining of a corresponding formalin fixed tumor sample.

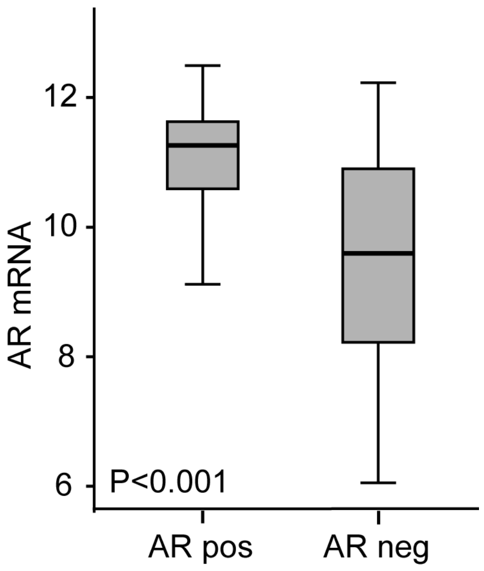

Supplementary Figure S1: There was a significant association between protein expression of AR evaluated by IHC and *AR* mRNA level.

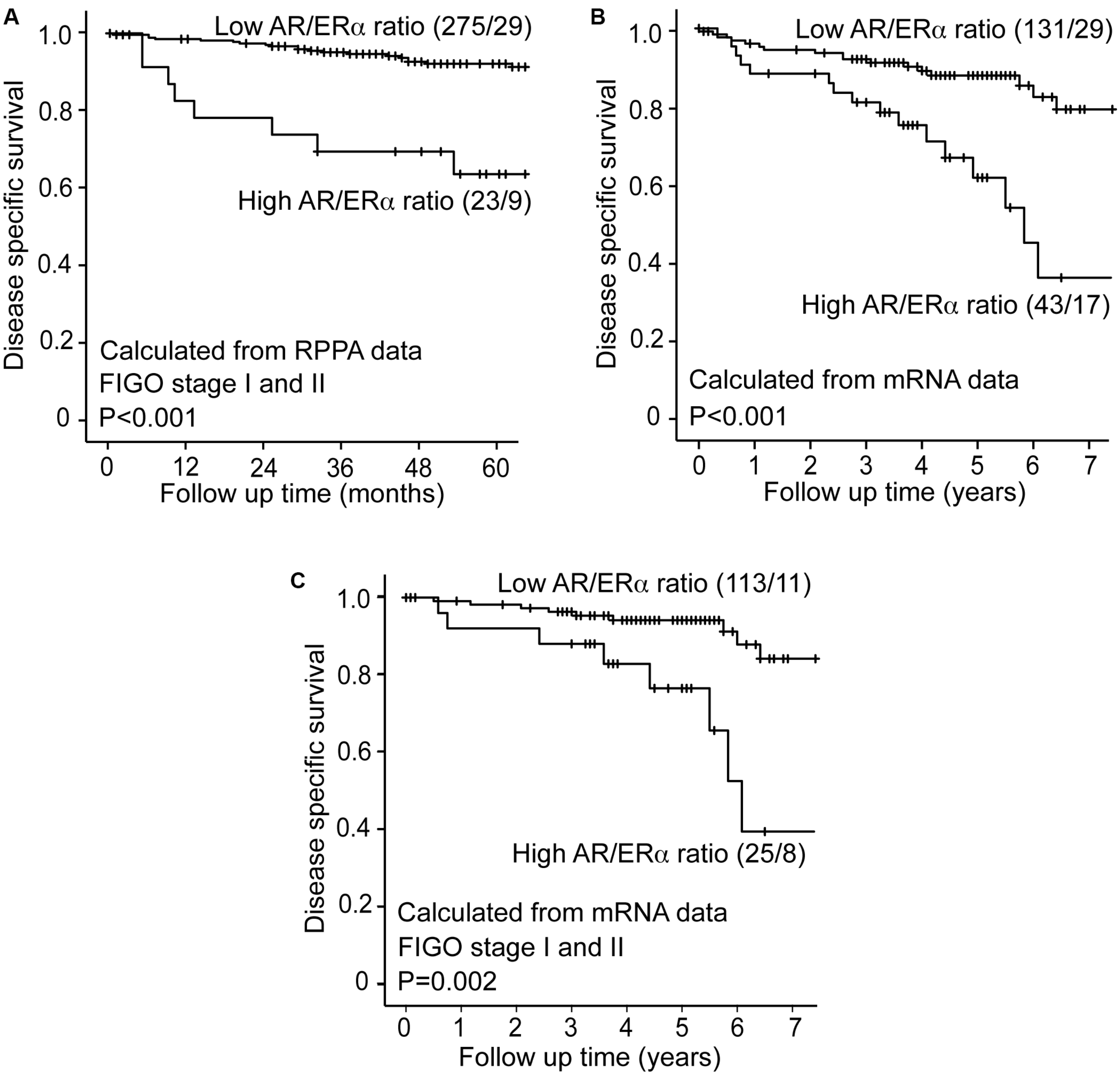

Supplementary Figure S2: High AR/ERα ratio is significantly associated with poor disease specific survival, within the subgroup of endometrial cancer patients with FIGO stage I/II (A). Patients with a high AR to ERα ratio calculated based on mRNA levels also demonstrate a particularly poor disease specific survival compared to patients with a low AR to ERα ratio both in the whole population (**B**) and in the subgroup with FIGO stage I/II (**C**).

Supplementary Table S1: Clinico-pathological characteristics for primary tumors with paired metastatic lesions (total number of patients: 142, total number of metastatic lesions: 298)

Variable	Primary tumor with paired metastatic lesions *n* (%)
Patient age	
< 66	54 (38)
≥ 66	88 (62)
FIGO-09 stage	
I–II	49 (35)
III–IV	93 (65)
Histologic type	
Endometrioid	76 (54)
Adenosquamous	2 (1)
Clear cell	10 (7)
Serous papillary	33 (23)
Carcinosarcoma	14 (10)
Undifferentiated/other	7 (5)
Histologic grade*	
Grade 1/2	52 (68)
Grade 3	24 (32)
Ploidy	
Diploid	63 (61)
Aneuploid	40 (39)
Number of metastases	
1	68 (48)
2	35 (25)
3	21 (15)
4	5 (3)
5	5 (3)
6	5 (3)
7	2 (1)
8	1 (2)

*Only endometrioid.

Supplementary Table S2: Clinico-pathological variables related to AR/ERα ratio

	AR/ERα ratio		
**Variable**	**Low *n* (%)**	**High *n* (%)**	***P*-value**
Age			0.3
< 66	169 (89)	20 (11)	
≥ 66	155 (86)	26 (14)	
FIGO-09 stage			< 0.001
I-II	275 (92)	23 (7)	
III-IV	49 (68)	23 (32)	
Histologic type			< 0.001
Endometrioid	284 (92)	25 (8)	
Adenosquamous	2 (67)	1 (33)	
Clear cell	5 (56)	4 (44)	
Serous papillary	24 (86)	4 (14)	
Carcinosarcoma	6 (40)	9 (60)	
Undifferentiated/other	3 (50)	3 (50)	
Histologic grade*			< 0.001
Grade 1/2	228 (95)	11 (5)	
Grade 3	51 (79)	14 (21)	
Metastatic nodes			< 0.001
Negative	234 (92)	21 (8)	
Positive	28 (64)	16 (36)	
Ploidy			< 0.001
Diploid	192 (92)	16 (7)	
Aneuploid	45 (75)	15 (25)	

*only endometrioid

Supplementary Table S3: GSEA (GO gene sets) showed that gene sets enriched in high AR to ER ratio group were associated with cell cycle regulation

Rank	Gene set	Size	NES	FDR (%)
1	CELL_CYCLE_PROCESS	174	–2.48	< 0.05
2	MITOTIC_CELL_CYCLE	139	–2.48	< 0.05
3	CELL_CYCLE_PHASE	155	–2.42	< 0.05
4	M_PHASE	102	–2.31	0.05
5	M_PHASE_OF_MITOTIC_CELL_CYCLE	78	–2.27	0.04
6	SPINDLE	37	–2.24	0.05
7	MITOSIS	76	–2.23	0.06
8	CELL_CYCLE_GO_0007049	288	–2.21	0.08
9	CHROMOSOME	118	–2.18	0.12
10	CHROMOSOMAL_PART	92	–2.15	0.19

Enriched in high AR to ER ratio group

Abbreviations: NES: normalized enrichment score, FDR: false discovery rate

Supplementary Table S4: Highest ranked compounds negatively correlated with gene expression profile of the group with high AR to ER ratio

Rank	Name of compound	Known target/function	*N*	*P*
1	Sirolimus	mTOR inhibitor	44	< 0.0001
2	Tanespimycin	HSP90 inhibitor	62	< 0.0001
3	LY-294002	PI3K inhibitor	61	< 0.0001
4	0175029-0000	CDK inhibitor	6	0.0002
5	Tretinoin	Retinoid, vitamin A analogue	22	0.0003
6	Sulconazole	Anti fungal drug	4	0.0004
7	Resveratrol	AR inhibitor	9	0.0007
8	Tranylcypromine	MAO inhibitor	5	0.002
9	Wortmannin	PI3K inhibitor	18	0.002
10	Geldanamycin	HSP90 inhibitor	15	0.003

N: number of instances the compound were tested in Connectivity Map.

Supplementary Table S5: Overview of methods applied and the number of patients and metastases investigated (number of cases overlapping with IHC)

Method applied	Number of hyperplasias	Number of primary tumors	Number of patients with available metastases	Total number of metastases
IHC AR	69	718	142	298
mRNA	18 (13)	174 (129)	31 (21)	42
RPPA		370 (306)		
